# The Relationship between Household Food Insecurity and Depressive Symptoms among Pregnant Women: A Cross Sectional Study

**Published:** 2020-04

**Authors:** Mahdi Khoshgoo, Omid Eslami, Mojtaba Khadem Al-Hosseini, Farzad Shidfar

**Affiliations:** Department of Nutrition, School of Public Health, Iran University of Medical Sciences, Tehran, Iran.

**Keywords:** *Depression*, *Food Insecurity*, *Pregnancy*, *Women*, *Socioeconomic Factors*

## Abstract

**Objective:** There is growing evidence suggesting that household food insecurity (HFI) is associated with adverse outcomes on mental health; however, limited evidence exists for pregnant women. This study was conducted to determine the relationship between HFI and depressive symptoms among a sample of pregnant women.

**Method** **:** This cross sectional study was performed on 394 pregnant women referring to the health centers located in Qom, Iran, from October 2017 to March 2019. HFI was evaluated using an 18-item US Household Food Security Survey Module. The Beck Depression Inventory-II questionnaire was applied to determine the severity of depressive symptoms. Logistic regression analysis was used to determine the factors associated with elevated depressive symptoms in the study population.

**Results: **The mean (± standard deviation) age of the study population was 28.59 ± 7.28 years. Almost 48% of participants were food insecure, and 37% experienced elevated levels of depressive symptoms during pregnancy. The prevalence of elevated depressive symptoms was significantly higher in food-insecure (P < 0.001) and unemployed (P = 0.02) women, while it was significantly lower in women with higher education levels (P < 0.001). In the adjusted model, it was revealed that HFI was significantly associated with the higher likelihood of having elevated depressive symptoms (OR = 3.31, 95% CI = 2.07, 5.29), while the higher level of education was negatively associated with the levels of depressive symptoms (OR = 0.40, 95% CI = 0.20, 0.79).

**Conclusion: **HFI was positively associated with the severity of depressive symptoms in a sample of pregnant women. Further studies are needed to confirm this finding. Meanwhile, routine screening of HFI for all pregnant women in the community health centers is recommended.

Household food insecurity (HFI) is a condition in which there is limited physical, social, or economic access to sufficient safe and nutritious food for an active and healthy life (1). HFI remains a serious public health concern in both developed and developing nations. According to the latest reports, the overall prevalence of HFI is estimated at 11.8% and 12% among American and Canadian households, respectively (2, 3). In developing countries, this figure was considerably higher, ranging between 30.4% up to 77.2% (4-6). In addition, the prevalence rate has been reported at 49% among Iranian households (7). In addition to the physical manifestations of HFI, such as the experience of a sharp pang of hunger, fatigue, or chronic illnesses, HFI could also be accompanied by several psychological conditions (8). There has been a growing interest in the relationship between HFI with depression in women, particularly in pregnant ones. 

Findings from several earlier studies have reported that HFI is positively associated with depressive symptoms in pregnant women (9-11). Pregnant women living in food-insecure households may experience difficulty accessing food resources or food preparation, and even may encounter financial problems, particularly those that are required to leave their workplace as the maternity leave. Moreover, they may face the challenge of how to allocate the limited food resources for their household to satisfy the dietary needs of the mother, fetus and the other family members.

Such negative experiences could create a stressful environment for food-insecure pregnant women, which has been closely linked with the risk of depression (10). Moreover, food-insecure women have lower diet quality, which is characterized by a lower intake of micronutrients, including vitamins and minerals as well as food sources (12). Such low-quality diets have been positively linked with depressive symptoms during the prenatal period, which may be due to absence of several major nutrients that are involved in mental health, such as folate, vitamin B-6, iron, selenium, zinc, and essential fatty acids (13, 14).

The overall prevalence of elevated depressive symptoms has been estimated at 41% among pregnant women in Iran (15). Evidence suggests that prenatal depression is positively associated with several adverse outcomes on maternal and infant health, such as postnatal depression (16, 17) low birth weight, and preterm birth (18, 19). Therefore, identifying factors that are associated with an elevated risk of depression during pregnancy is necessary. To our knowledge, no study has been conducted to evaluate the association between HFI and depression among the Iranian pregnant women. Iran is located in the Middle East and North Africa (MENA) region, with a severe food insecurity prevalence of 11%, this country has been recognized as one of the most food-insecure regions in the world (6). Over the last 3 decades, Iran has made a remarkable improvement in reducing food insecurity, so that it has the greatest reduction rate in global hunger index ranking in the Middle East (20). Nevertheless, HFI still remains a public health challenge. According to the latest national surveys, almost 14 provinces have been categorized to be moderate to severe food insecure; also, based on the global food security risk index, Iran has been reported as a country with moderate risk of food insecurity (21). Thus, the present study was conducted to evaluate the relationship between HFI with depressive symptoms among pregnant women in Iran.

## Materials and Methods

***Participants***

The present cross sectional study included 394 pregnant women referring to the health centers in Qom, northcentral of Iran, between October 2017 to March 2019. For sampling, Qom was divided into 5 geographical regions, including north, south, east, west, and center. Of each district, 3 health centers were selected using the randomized cluster sampling method, and convenience sampling method was used to choose participants in each center. Inclusion criteria were as follow: (1) no physician-diagnosed mental disorders; (2) not taking antipsychotic medications; and (3) no chronic diseases or pregnancy complications, such as gestational diabetes mellitus, preeclampsia, or anemia. The objectives of the study were explained to the participants and those who wished to participate signed a written informed consent. The protocol of this study was approved by the Ethics Committee of Iran University of Medical Sciences, Tehran, Iran.

***Data Collection***

Sociodemographic characteristics of study participants, including age, education level, occupation, and the number of children, were collected by a researcher-made questionnaire using face-to-face interviews. Also, information on pregnancy trimester and prepregnancy body mass index (BMI) was obtained from health records. 

***HFI Assessment***

We used the Persian version of the Household Food Security Survey Module (HFSSM) to assess the status of food security of households over the last 12 months. The validity of the Persian version of the questionnaire has already been confirmed in an earlier study; also, it showed good reliability in the sample of Iranian women, with a Cronbach's alpha of 0.72 (22, 23). The questionnaire consisted of 2 sections comprising a total of 18 items; the first section was filled for all households (10 questions), while the second section was just completed for households who had a child under the age of 18 years (8 questions). In each question, the lack of money to meet the food needs was considered as the reason for the condition or behavior, so they were not influenced by nonfinancial problems, such as hunger due to voluntary dieting/ fasting or food reluctance. The questions covered a wide range of food-related behaviors, experiences, and conditions due to financial limitation: worrying about running out of food, cutting/skipping meals, inability to provide a balanced meal, not eating for a whole day, losing weight/being hungry due to insufficient food, relying on low-cost food, and failing to provide enough food supply for the household. A score of 1 was assigned to an item in the questionnaire if the participant’s response affirmatively included the following answers: yes, often true, sometimes true, almost every month, some months but not every month; Otherwise, negative responses, such as no, never true, and only 1 or 2 months, received no score (score 0). Then, the affirmative responses were summed up and the status of food security was determined based on the total scores obtained from the questionnaire (24), which is presented in Table 1. 

***Assessment of Depressive Symptoms***

The severity of depressive symptoms in pregnant women was evaluated using the Persian version of the Beck Depressive Inventory-II (BDI-II) questionnaire, which has been validated among the Iranian population in a study by Ghassemzadeh et al (25). It contains 21 self-evaluation items that investigate the experiencing of different symptoms of depression over the past 2 weeks. Each item is assigned a score, ranging from 0 to 3, and the severity of depressive symptoms is determined based on the sum of the scores, ranging from 0 to 63 points. A total score of 0 to 13, 14 to 17, 18 to 19, and 23 points and above were considered as having no/ minimal, mild, moderate, and severe depressive symptoms, respectively (26).

***Statistical Analysis***

SPSS software version 22 (IBM Corp., Armonk, NY, USA) was used for statistical analysis. Chi-square test was used to compare the food security status, sociodemographic, and midwifery characteristics between those with none/mild depressive symptoms and those with elevated depressive symptoms. Univariate analysis and logistic regression model were used to determine the variables that were associated with elevated depressive symptoms in the study population. The goodness-of-fit of the regression model was checked using the Hosmer–Lemeshow test. Data were expressed as frequency and percentage, odds ratio (OR), and 95% confidence intervals (CI). Significance level was defined as a p value less than 0.05.  

## Results

A total of 394 eligible pregnant women participated in this study and were included in the final analysis. The mean (± standard deviation (SD)) age of the study population was 28.59 ± 7.28 years. About 5% of the women had no academic education (n = 18; 4.6 %), while over one-third were graduated with a high school degree. Also, almost 80% of the participants were unemployed. In terms of midwifery characteristics, about 40% were primiparous, 46.2% had a bodyweight within the normal ranges before pregnancy, and over 30% were in the third trimester of their pregnancy. Over half of the population were categorized as food secure, while the prevalence of mild, moderate, and severe food insecurity was 27.2% (n = 107), 12.4% (n = 49), and 7.9 % (n = 31), respectively. The mean (± SD) score of BDI-II was 13.35 ± 11.96. Also, assessment of depressive symptoms in the study sample revealed that 62.2% (n = 245) had no or minimal depressive symptoms, while the prevalence of mild, moderate, and severe depressive symptoms was 16.8% (n = 66), 9.6% (n = 38), and 11.4% (n = 45), respectively. 

Table 2 compares the sociodemographic and midwifery characteristics between participants with no depression and those with elevated depressive symptoms. The prevalence of elevated depressive symptoms was higher in food-insecure women compared to the food-secure (68.5% vs 31.5%, respectively, P < 0.001). Also, women with higher education had a lower prevalence of elevated depressive symptoms compared to those who had not attained a high-school degree (21.5% vs 38.9% for elevated depressive symptoms, P < 0.001). Moreover, unemployed women had higher percentages of elevated depressive symptoms rather than the employed (85.2% vs 14.8%, respectively, P = 0.02). 

The results of univariate and multiple regression analyses are presented in Table 3. In the crude analysis, higher level of education (OR = 0.32, 95% CI = 0.19, 0.55) and being employed (OR = 0.54, 95% CI = 0.31, 0.93) were significantly associated with lower odds of elevated depressive symptoms. While being food insecure (OR = 4.08, 95% CI: 2.64, 6.30), as well as having 2 or more children (OR = 1.69, 95% CI: 1.04, 2.76) were significantly associated with higher odds. However, when variables were entered into the adjusted model, the association remained only significant for higher education level (OR = 0.40, 95% CI= 0.20, 0.79) and being food insecure (OR = 3.31, 95% CI= 2.07, 5.29). The results of Hosmer-Lemeshow goodness-of-fit test showed a good calibration for regression model (Chi-square 7.06, degrees of freedom = 8; P value = 0.53).

## Discussion

This was the first study to investigate the association between HFI with depressive symptoms among pregnant women in Iran. Also, to our knowledge, only 1 study conducted by Kazemi et al (27) has examined the prevalence of food insecurity in Iranian pregnant women. They reported that the prevalence of HFI was about 44% among pregnant women in Qazvin, northwest of Iran. The prevalence of HFI in our study sample was nearly 48%, which is comparable to the prevalence rate reported by Kazemi et al (27).

To date, a few numbers of studies have assessed the relationship between food insecurity with depression in pregnant women. In this study, we found that women living in households with food insecurity had a higher likelihood of experiencing elevated depressive symptoms during pregnancy. This was in line with the results of a study by Laraia et al (11) among 606 American pregnant women, in which the depressive symptoms score was positively associated with HFI ( OR = 1.87, 95% CI = 1.40, 2.51). Also, Hromi-Fiedler et al (10) demonstrated that among 135 American pregnant women, those with food insecurity had significantly higher odds of experiencing increased levels of depressive symptoms compared to their food-secure counterparts (OR = 2.59, 95% CI = 1.03–6.52). Moreover, a study by Natamba et al, which was conducted on 403 pregnant women in Uganda, showed that food insecurity was positively associated with the severity of depressive symptoms (28). Similarly, Abrahams et al (9) found that in a group of pregnant women in South Africa (n = 376), food-insecure participants had 5 times higher risk of having major depressive episodes than the food-secure ones.

Despite the consistent results, we observed a considerable heterogeneity between the studies in terms of the type of screening tools for assessment of food insecurity and depression. For example, Laraia et al, Hromi-Fiedler et al, and Abrahams et al evaluated food insecurity at the household level using 18-item, 15-item, and 6-item HFSSM, respectively (9-11). However, Natamba et al assessed food insecurity at the individual level using the 9-item individually focused FI access scale (IFIAS). Also, the cutoffs for diagnosis of food-insecure participants varied between studies; Hromi-Fiedler et al had classified pregnant women as food-insecure if they responded affirmatively to any question in HFSSM (10), while in Abrahams et al study, participants, with a score ranging between 2 to 6, were considered to be food-insecure (9). In terms of depression screening, 3 studies (10, 11, 28) used the 20-item Center for Epidemiological Studies Depression (CES-D) scale, which unlike the BDI-II, investigates the severity of depressive symptoms based on the number of days of experiencing the related symptoms during the past week. Also, the demographic and socioeconomic status of participants varied between studies. The study population in the studies conducted by Hromi-Fiedler et al and Laraia et al comprised of pregnant women who had participated in or were eligible for the Special Supplemental Nutrition Program for Women, Infants, and Children (WIC) program, a federal assistance program for improving the health of low-income pregnant women with household income below 185% of the federal poverty line (10). Thus, these differences should be considered when the interpretation of the results. 

In the present study, we found that education level was inversely associated with severity of depressive symptoms in pregnant women. Such an inverse association was also reported in several earlier studies conducted in pregnant women (29-31). Limited literacy has been linked with poor self-efficacy, low self-esteem, and feelings of worthlessness, guilt or shame; such characteristics are also common in individuals with depression (32). In addition, a meta-analysis showed a 3% decrease in odds of having depression for each additional year of education (33). It is hypothesized that literacy could decrease depressive symptoms through improving self-efficacy (34). 

However, other studies could not find an association between education and depression in pregnant women. In a study of 258 pregnant women living in Turkey, there was no significant correlation between the mean values of depression score and educational level (35). Also, in another study by Jarahi et al among a sample of 300 Iranian pregnant women, no significant difference was observed between the depressed and nondepressed population in terms of their level of education (36). The conflicting results between studies may be due to the differences in the methodology of studies, including the baseline socioeconomic status of participants, sample size, the definition of low versus high education level, the type of questionnaires used for assessment of depressive symptoms, and the cutoffs for diagnosis of elevated depressive symptoms.

## Limitation

Several limitations of this study should be acknowledged. First, because of the cross sectional design of the study, it was not possible to conclusively determine the direction of the relationship that whether being food secure is a risk factor for the development of depression or being depressed is accompanied by developing food insecurity in the pregnant women. Second, the study population consisted of women referring to urban health centers, most of whom had a graduate degree and were within the normal ranges of weight and had no pregnancy complications. Whether the finding of this study is generalizable to pregnant women with different sociodemographic status remains unclear. Third, we did not collect the data on dietary intake of study participants, which could provide better conclusive evidence regarding the association of HFI and depressive symptoms.

## Conclusion

In summary, the present study showed a positive association between HFI and the severity of depressive symptoms in a sample of pregnant women in Iran. Based on the findings, it is recommended that screening for HFI and depressive symptoms be done routinely in health centers. Moreover, interventional studies are needed to assess the efficacy of supplemental nutrition programs related to HFI on mental health in this at-risk group.

**Table 1:** Classification of Household Food Security Status in the Study Population

**Food Security Status**	**Number of Positive Answers**	
	**Households without children under ** **18 years (total score: 10 points)**	**Households with children under 18 ** **years (total score: 18 points)**
Food secure	0-2	0-2
Food insecure without hunger	3-5	3-7
Food insecure with moderate hunger	6-8	8-12
Food insecure with severe hunger	9-10	13-18

**Table 2:** Sociodemographic and Midwifery Characteristics of Study Participants

				**Level of Depressive Symptoms**				
**Variable**		**Total** **(n = 394)**		**None / Minimal** **(n = 245)**		**Elevated** **(n = 149)**		***P *** **value ** †
		N	%	N	%	N	%	
Age (years)								
	< 24	121	30.7	77	31.4	44	29.5	0.28
	24 to 34	189	48	122	49.8	67	45	
	≥ 34	84	21.3	46	18.8	38	25.5	
Education								
	< High school	119	30.2	61	24.9	58	38.9	< 0.001 *
	High school graduated	140	35.5	81	33.1	59	39.6	
	Higher	135	34.3	103	42	32	21.5	
Occupation								
	Unemployed	313	79.4	186	75.9	127	85.2	0.02 *
	Employed	81	20.6	59	24.1	22	14.8	
Number of children								
	None	159	40.4	105	42.9	54	36.2	0.06
	1	117	29.7	77	31.4	40	26.8	
	2 or more	118	29.9	63	25.7	55	36.9	
Pregravid BMI								
	Underweight	51	12.9	29	11.8	22	14.8	0.52
	Normal weight	182	46.2	118	48.2	64	43	
	Overweight or obese	161	40.9	98	40	63	42.3	
Pregnancy trimester								
	1^st^	132	33.5	73	29.8	59	39.6	0.13
	2^nd^	139	35.3	91	37.1	48	32.2	
	3^rd^	123	31.2	81	33.1	42	28.2	
Food security status ‡								
	Food secure	207	52.5	160	65.3	47	31.5	< 0.001 *
	Food insecure	187	47.5	85	34.7	102	68.5	

Abbreviation: BMI, body mass index.

† Obtained from chi-square test.

* Significant at the level of *P* < 0.05.

‡ The food insecure group was comprised of pregnant women without/ with moderate/ with severe hunger.

**Table 3:** Factors Related to the Elevated Depressive Symptoms among Pregnant Women Using Univariate and Logistic Regression Analysis (n = 394)

		**Crude**			**Adjusted ** †			
**Variable**		**OR**	**95 % CI**		**β**	**OR**	**95 % CI**	
			Lower	Upper			Lower	Upper
Age								
	< 24	1				1		
	24 to 34	0.96	0.59	1.54	0.30	1.35	0.69	2.63
	≥ 34	1.44	0.82	2.54	0.27	1.31	0.53	3.19
Education								
	< High school	1				1		
	High school graduated	0.76	0.46	1.25	- 0.15	0.85	0.49	1.47
	Higher	0.32 *	0.19	0.55	- 0.89	0.40 *	0.20	0.79
Occupation								
	Unemployed	1				1		
	Employed	0.54 *	0.31	0.93	- 0.15	0.85	0.45	1.63
Number of children								
	None	1				1		
	1	1.01	0.61	1.67	- 0.06	0.93	0.50	1.74
	2 or more	1.69 *	1.04	2.76	0.09	1.09	0.52	2.28
Pregravid BMI								
	Underweight	1				1		
	Normal weight	0.71	0.38	1.34	- 0.17	0.83	0.41	1.69
	Overweight or obese	0.84	0.44	1.60	- 0.005	0.99	0.48	2.04
Pregnancy trimester								
	1^st^	1				1		
	2^nd^	0.65	0.40	1.06	- 0.439	0.645	0.377	1.103
	3^rd^	0.64	0.38	1.06	- 0.436	0.647	0.372	1.123
Food security status ‡								
	Food secure	1				1		
	Food insecure	4.08 *	2.64	6.30	1.19	3.31 *	2.07	5.29

Abbreviations: OR, odds ratio; CI, confidence interval; BMI, body mass index.

† Results were obtained from logistic regression analysis using the severity of depressive symptoms as the dependent variable and all respective covariates as independent variables.

* Significant at the level of *P *< 0.05.

‡ Participants classified as food insecure without hunger, as well as with moderate and severe hunger were merged into the food insecure group.
